# Dose-Dependent Effects of Supplementing a Two-Strain *Bacillus subtilis* Probiotic on Growth Performance, Blood Parameters, Fecal Metabolites, and Microbiome in Nursery Pigs

**DOI:** 10.3390/ani14010109

**Published:** 2023-12-28

**Authors:** Karyn A. Duddeck, Tiffany E. Petersen, Haley J. Adkins, Alexandra H. Smith, Samantha Hernandez, Seth J. Wenner, Dan Yao, Chi Chen, Wenli Li, Priscila Fregulia, Anna Larsen, Young Dal Jang

**Affiliations:** 1 Department of Animal and Food Science, University of Wisconsin-River Falls, River Falls, WI 54022, USA; 2 The ScienceHearted Center, Arm & Hammer Animal and Food Production, Waukesha, WI 53186, USA; 3 Department of Animal Science, University of Minnesota, St. Paul, MN 55108, USA; 4 United States Department of Agriculture-Agricultural Research Service, US Dairy Forage Research Center, Madison, WI 53706, USA; 5 Oak Ridge Institute for Science and Education, Oak Ridge, TN 37830, USA; 6 Department of Animal and Dairy Sciences, University of Wisconsin-Madison, Madison, WI 53706, USA; 7 Department of Animal and Dairy Science, University of Georgia, Athens, GA 30602, USA

**Keywords:** weaning, pigs, growth, *Bacillus subtilis*, supplementation level, short-chain fatty acids

## Abstract

**Simple Summary:**

Postweaning diarrhea due to pathogenic *Escherichia coli* is a common issue in swine production. Specific strains of *Bacillus subtilis* have been shown to reduce the incidence and severity of diarrhea and thereby improve the growth of nursery pigs. The current study aims to demonstrate the effect of *B. subtilis* supplementation of two strains selected to reduce the effects of pathogenic *E. coli* to nursery pig diets on growth performance, blood biochemicals, fecal metabolites, and microbiome. *B. subtilis* supplementation to nursery diets at 1.875 × 10^5^ CFU/g diet improved early postweaning growth performance, increased blood glucose levels, and fecal short-chain fatty acid production, which is beneficial for the gut health and development for nursery pigs. But a higher dose of *B. subtilis* did not affect pig growth and fecal short-chain fatty acid production. These results suggest that *B. subtilis* supplementation to nursery pig diets at 1.875 × 10^5^ CFU/g diet is beneficial for growth and gut health, whereas a higher dose of the probiotic may not be as effective as the recommended level.

**Abstract:**

This experiment was conducted to evaluate the effects of dietary supplementation level of a two-strain *Bacillus subtilis* probiotic on growth performance, blood parameters, fecal metabolites, and microbiome in nursery pigs. A total of 54 weaned piglets were allotted to three treatments in three replicate pens with six pigs/pen for a 28 d feeding trial. The treatments were as follows: control: no probiotic supplementation; Pro1x: *B. subtilis* supplementation at 1.875 × 10^5^ CFU/g diet; and Pro10x: *B. subtilis* supplementation at 1.875 × 10^6^ CFU/g diet. Body weight at d 14 postweaning (*p* = 0.06) and average daily gain for d 0 to 14 postweaning (*p* < 0.05) were greater in the Pro1x treatment than in the other treatments. Blood glucose levels were greater in both probiotic treatments than in the control treatment at d 14 postweaning (*p* < 0.05). In the fecal short-chain fatty acid (SCFA) concentrations, the butyrate concentrations were greater in the Pro1x treatment than in the other treatments (*p* < 0.05), and the acetate, propionate, and total SCFA concentrations were greater in the Pro1x treatment than in the Pro10x treatment (*p* < 0.05). The beta diversity of fecal microbiome composition at d 14 postweaning based on Unweighted Unifrac analysis was dissimilar between the Pro1x and Pro10x treatments (*p* < 0.05). In conclusion, dietary *B. subtilis* supplementation of two strains selected to reduce effects of pathogenic *Escherichia coli* to nursery diets at 1.875 × 10^5^ CFU/g diet improved the growth rate in the early postweaning period, increased fecal SCFA concentrations and altered the fecal microbial community composition. A higher dose of *B. subtilis* did not improve the performance parameters over those of the control piglets.

## 1. Introduction

Weaning is a major stressful event for young piglets, as it involves sudden changes in diet from milk to solid form as well as their social and living environments. Newly weaned pigs, due to insufficient immunoprotection, are susceptible to pathogenic-bacterial-infection-elicited gastrointestinal disorders, resulting in diarrhea, growth retardation, and early death [1]. Antibiotics have been widely used in nursery pig diets to reduce the occurrence of diarrhea and improve the postweaning growth rate. However, because of concerns of antibiotic resistant bacteria resultant from using antibiotics in swine diets, researchers have been investigating the efficacy of alternatives to antibiotics that can be used in nursery pig diets. 

Probiotics have been widely used in swine diets and are beneficial to improve the intestinal health of postweaning piglets by suppressing pathogenic bacteria, enhancing beneficial bacterial proliferation, stimulating the immune system, degrading toxins, producing short-chain fatty acids (SCFA) that are potential energy sources for piglets [2], and modulating the bile acid profile of pigs [3]. As the gut is responsible for the first-line defense against bacterial infection and ingested toxic substances, a healthy gut environment with a well-balanced microbial community is important to protect weaning piglets from gut dysbiosis and diarrhea in the nursery period [2]. It has been reported that dietary supplementation of probiotics in swine diets, especially for nursery pigs, could improve the gut microbiota, gut immune system, and nutrient utilization so that it could reduce weaning stress and improve growth and survival rate [4,5,6,7]. 

*Bacillus subtilis* is a spore-forming bacteria that has been shown to improve growth performance and immunity, reduce postweaning diarrhea, and modulate the gut microbiota of weaning pigs [6,7,8]. *Bacillus subtilis* is known to consume oxygen in the gastrointestinal tract, produce several enzymes that provide a positive environment for lactic acid-producing bacteria, be resistant to high temperature, and improve digestive enzyme activities for degrading carbohydrates, protein, lipids, and fiber [9,10]. Several previous studies have demonstrated that *B. subtilis* supplementation in nursery diets improved growth performance, gut immunity, and microbiota under normal and disease-challenged conditions [11,12,13,14]. Therefore, *B. subtilis* has been widely used in diets for weaning pigs to reduce postweaning diarrhea, enhance gut integrity, immunity, and barrier functions, and thereby improve postweaning growth performance.

However, the efficacy of probiotic products is dependent on numerous factors, such as the species and strains of the microbe, product formulation (single- or multi-strain probiotics), the health condition being targeted, as well as the dose of the product administered [8]. Although the definition of probiotics is that they are live microorganisms beneficial to the health of the host when administered in adequate amounts, there is limited information about the effect of the dose of dietary probiotics on the growth performance, blood parameters, fecal metabolites, and microbiome of nursery pigs. Therefore, the objective of this study was to evaluate the effects of dietary supplementation with low and high levels of a two-strain *B. subtilis* probiotic product developed to reduce the effects of F18 enterotoxigenic *Escherichia *coli**, a common cause of diarrhea in piglets postweaning, on the growth performance, blood parameters, fecal metabolites, and microbiome of nursery pigs. The hypotheses of the study were that (1) *B. subtilis* supplementation at the level recommended by the manufacturer could improve growth performance and affect the fecal metabolites (SCFA and bile acids) and microbiome of pigs, and (2) an overdose of *B. subtilis* could be detrimental to newly weaned pigs.

## 2. Materials and Methods

### 2.1. Animal Care 

All procedures used in this study were approved by the Institutional Animal Care and Use Committee of the University of Wisconsin-River Falls (UWRF; Protocol # 20-21-41778). The experiment was conducted in the nursery facility at Mann Valley Farm at UWRF (River Falls, WI, USA).

### 2.2. Animals, Housing, and Treatments

A total of 54 piglets (initial body weight (BW): 9.1 ± 1.3 kg; Yorkshire × Yorkshire, Yorkshire × Duroc, Yorkshire × Duroc × Duroc) weaned at 26.9 ± 2.0 d of age were allotted into 3 treatments in 3 replicate pens with 6 pigs per pen (3 barrows and 3 gilts) with a total of 9 pens based on body weight (BW), breed, sex, age, and littermate for a 28 d growth trial. All piglets were housed in raised-deck nursery pens (1.32 × 1.63 m^2^) with plastic or woven-wire flooring in an environmentally controlled nursery at the UWRF Mann Valley Farm facility. No creep feed was provided during the lactation period. The treatments were as follows: (1) control: no probiotic supplementation, (2) Pro1x: 3.75 × 10^8^ CFU/g *B. subtilis* supplementation (0.05% supplementation level resulting in 1.875 × 10^5^ CFU/g diet; the level recommended by the manufacturer), and (3) Pro10x: 3.75 × 10^9^ CFU/g *B. subtilis* supplementation (0.05% supplementation level resulting in 1.875 × 10^6^ CFU/g diet). The probiotic product was obtained from Arm & Hammer Animal and Food Production (The ScienceHearted Center, Waukesha, WI) and consisted of a blend of two strains selected to inhibit 35 pathogenic F18 *E. coli* strains often implicated in piglet diarrhea.

### 2.3. Experimental Diets

All piglets were fed ad libitum corn–soybean meal-based diets formulated to meet or exceed the nutrient requirement estimates of the NRC [15] with free access to water and feed for the 28 d of the entire experimental period. Two diet phases were included: d 0–14 (Phase 1), and d 14–28 (Phase 2) postweaning (Table 1). 

The probiotic product was supplemented in the diets at the assigned levels by replacing corn. For mixing experimental diets, a single batch of the basal diet was mixed without the probiotic product to prevent differences in the non-treatment components of the diets. Then, the basal diet was divided into 3 fractions. One fraction was mixed with an additional 0.05% corn for the control diet without probiotic supplementation, and 0.05% of each probiotic product was added to the other fraction to create each probiotic treatment diet. Feed samples for each phase were collected from diet mixing and analyzed for *Bacillus* spp. count at the Arm & Hammer Animal and Food Production Laboratory (Waukesha, WI, USA), following the procedures outlined in the Bacteriological Analytical Manual [16]. Briefly, feed samples were diluted 1:10 in 0.1% peptone, masticated, and plated onto selective media, and the result is presented in Table 2.

### 2.4. Growth Performance Measurement and Sample Collection

The body weight of each pig and feed consumption were recorded at d 0 (study initiation), d 14, and 28 postweaning for the calculation of average daily gain, average daily feed intake, and gain-to-feed ratio. The fecal score was recorded every day for the entire experimental period using a 4-point scale fecal score system (1 = normal; 2 = soft, looser than normal feces, slight diarrhea; 3 = moderate diarrheic feces; and 4 = liquid, severe diarrhea) by observing individual pigs in each pen and assessing signs of stool consistency in the pen.

Blood samples from 2 pigs (average body weight) per pen were collected from the jugular vein into K_3_-EDTA tubes at d 0 (initial), 14 and 28 postweaning. Whole blood samples were analyzed for hematocrit, creatinine, and glucose levels. For hematocrit level determination, two 75 mm sodium-heparinized capillary tubes (Jorgensen Laboratories Inc., Loveland, CO, USA) were filled with blood from each blood sample and then packed with clay prior to being put in the micro-hematocrit centrifuge (UNICO, Dayton, NY, USA). After the samples were spun down at 10,000 g for 6 min at room temperature and the plasma and red blood cells were separated, the capillary tubes were placed on a microhematocrit reader (Jorgensen Laboratories Inc., Loveland, CO, USA) and the hematocrit level was determined in duplicate by two trained observers. Blood glucose and creatinine levels were analyzed using the iSTAT blood analyzer (Abbott AG, Baar, Switzerland). 

Fecal samples were collected from 2 representative pigs in each pen (average body weight) via rectal palpation at d 14 and 28 postweaning for fecal SCFA and bile acid analysis. All fecal samples were flash-frozen in liquid nitrogen and stored at −80 °C for further analysis. 

### 2.5. Fecal Metabolites Analysis 

Fecal samples were prepared by mixing with 50% aqueous acetonitrile in a 1:10 (*w*/*v*) ratio and then centrifuging at 18,000× *g* for 10 min to obtain fecal extract supernatants. Fecal extracts were separated using a BEH C18 column (Waters, Milford, CT, USA) in an Acquity^TM^ ultraperformance liquid chromatography system (Waters) and then detected in a Xevo-G2-S quadrupole time of flight mass spectrometer (QTOFMS) system (Waters). To detect SCFA, fecal extracts were first derivatized using 2-hydrazinoquinoline (HQ) and then detected in the positive mode in the liquid chromatography–mass spectrometry (LC-MS) analysis [17], while bile acids were detected in the negative mode in the LC-MS analysis. The conditions of LC-MS analysis, including mobile phase, and the parameters of MS detection have been described previously [18]. Mass chromatograms and mass spectral data were acquired and processed using the MassLynx^TM^ software V4.2 (Waters) in a centroided format. Individual compound concentrations were determined by calculating the ratio between the peak area of compound and the peak area of the internal standard and fitting with a standard curve using the Quanlynx^TM^ software V4.2 (Waters).

### 2.6. DNA Extraction, Library Preparation and Sequencing

Fecal samples were collected from 2 representative pigs in each pen (average body weight) via rectal palpation at d 14 and 28 postweaning for fecal microbiome analysis. Total DNA was extracted from 250 mg of swine feces using Qiagen RNeasy Power microbiome kit according to the manufacturer’s instructions (Qiagen, Germany). DNA quantity was checked using Qbit dsDNA assay (Q32851, ThermoFisher, Waltham, MA, USA). For 16S rRNA library preparation, 35 ng of total DNA was used as the input for each sample, using the xGen Amplicon 16S panel library kit (IDT, Integrated DNA Technologies, Coralville, IA, USA). This kit targets V1–V9 region of the 16S rRNA. All libraries were prepared according to the manufacturer’s instructions, except the multiplex PCR thermal cycler program, which was carried out as follows: 98 °C for 30 s; 10 cycles of 98 °C for 10 s, 63 °C for 5 min and 65 °C for 1 min; 24 cycles of 98 °C for 10 s and 64 °C for 1 min; 65 °C for 1 min; 4 °C hold. Quantification of prepared libraries was performed using a Kapa Library Quantification kit (KK4873, Kapa systems, Roche, Switzerland) with a QuantStudio 5 qPCR instrument (ThermoFisher, Waltham, MA, USA) with an expected fragment size of 475 bp, following the manufacturer’s instructions. Using the concentration generated via the Kapa quantification kit, sequencing pooling was prepared using the pooling calculator by Illumina (https://support.illumina.com/help/pooling-calculator/pooling-calculator.htm, accessed on 20 March 2023). Pooled libraries were first sequenced using a MiSeq Nano 300 cycle kit (Illumina Inc., San Diego, CA, USA) to obtain an individual index ratio for each sample in the pool. To ensure equal depth of sequencing of all the samples in the pool, the pooling volume was further normalized according to the index ratios. Finally, normalized, pooled libraries were sequenced on the Illumina MiSeq platform to generate 2 × 150 bp paired-end reads (Illumina Inc., San Diego, CA, USA). 

### 2.7. Statistical Analysis and Amplicon Sequencing Data Analysis

All data were analyzed via ANOVA for a randomized complete block design using PROC MIXED of SAS (version 9.4; SAS Inst. Inc., Cary, NC, USA). The treatment was used as a fixed effect, and the replicate was used as a random effect. A pen in the feeding trial was used as the experimental unit for body weight, average daily gain, average daily feed intake, gain-to-feed ratio, and fecal score, and an individual pig was used as the experimental unit for blood parameters and fecal SCFA and bile acid concentrations and microbiome. Statistical outliers were identified using the Grubbs test outlier calculator (GraphPad Software, San Diego, CA, USA) and excluded from the data analysis (1 from Pro1x and 1 from Pro10x) due to negative growth rate until 2 weeks postweaning. The feed intake data for those pigs were adjusted as described by Lindemann and Kim [19]. Least squares means were separated using the PDIFF option of SAS. Statistical differences were considered significant at *p* < 0.05 and tendency at *p* < 0.10. 

Microbial sequence data were analyzed using Quantitative Insights Into Microbial Ecology 2 (QIIME 2) version 2021.11 [20] following the default method in the QIIME2 website. In brief, the data were demultiplexed and the reads were quality-filtered using the Divisive Amplicon Denoising Algorithm (DADA2) plugin implemented in QIIME2. The sequences were merged, and chimeric sequences were removed before the generation of a table of amplicon sequencing variants (ASV) [21]. Representative sequences were aligned to the SILVA (Silva 138 99% OTUs full-length sequences).

To analyze microbial diversity, sequence counts were standardized by rarefying them to the same number of sequences (the smallest sampling size). To investigate the alpha diversity metrics, Pielou’s evenness, Shannon’s entropy, and Faith’s PD were calculated. To investigate the beta diversity metrics, the Bray–Curtis dissimilarity index, Jaccard index, and weighted UniFrac distance were calculated. Dissimilarity and distance between the fecal microbiota and the variables of the study (period, treatment, and the interaction of period × treatment) were tested using Unweighted UniFrac distance matrices.

## 3. Results

The actual count of *Bacillus* spp. in the experimental diets met or exceeded the target supplementation level in each experimental diet, confirming the experimental diets were mixed properly for the experiment (Table 2), except for the *Bacillus* spp. count in the Pro10x diet in Phase 2, which was greater than expected. A small amount of feed sample from a large feed batch was used in the analysis, which could result in a variation in the analysis. With a greater bacterial count in all diets, it is assumed that the pigs consumed the probiotic over the anticipated level from the diets. 

In growth performance, the BW at d 14 postweaning (*p* = 0.055; 11.98, 12.73, and 12.21 kg for control, Pro1x and Pro10x treatments, respectively) and average daily gain for d 0 to 14 postweaning (*p* < 0.05; 0.206, 0.259, and 0.222 kg/d for control, Pro1x and Pro10x treatments, respectively) were greater in the Pro1x treatment than in the control and Pro10x treatments, although there was no significant difference in growth performance at d 28 postweaning (*p* = 0.96; 20.37, 20.50, and 20.38 kg for control, Pro1x and Pro10x treatments, respectively). There was no significant difference in growth performance between the control and Pro10x treatments. Overall average daily feed intake (*p* = 0.54; 0.652, 0.678, and 0.691 kg/d for control, Pro1x and Pro10x treatments, respectively), gain-to-feed ratio (*p* = 0.53; 0.618, 0.603, and 0.587 kg/kg for control, Pro1x and Pro10x treatments, respectively), and fecal score (*p* = 0.75; 1.62, 1.56, and 1.56 for control, Pro1x and Pro10x treatments, respectively) had no significant differences among dietary treatments. 

Although the blood hematocrit and creatinine levels (Table 3) were not different among all dietary treatments, the blood glucose levels were greater in both probiotic treatments than in the control treatment at d 14 postweaning (*p* < 0.05).

In the fecal SCFA concentrations (Table 4) at d 28 postweaning, the butyrate and isovalerate concentrations were greater in the Pro1x treatment than in the control and Pro10x treatments (*p* < 0.05), and the acetate, propionate, and total SCFA concentrations were greater in the Pro1x treatment than in the Pro10x treatment, with the intermediate value being found in the control treatment (*p* < 0.05).

There were no significant differences in fecal bile acid concentrations on both d 14 (Table 5) and 28 (Table 6) postweaning. However, both probiotic treatments tended to decrease the proportion of glycohyocholic acid to total bile acid (*p* = 0.09) on d 14 postweaning. Moreover, the Pro10x treatment tended to increase the ratio between hyodeoxycholic acid, the most abundant secondary bile acid, and hyocholic acid, the precursor primary bile acid of hyodeoxycholic acid, on d 14 postweaning (*p* = 0.08). 

**Figure 1:**
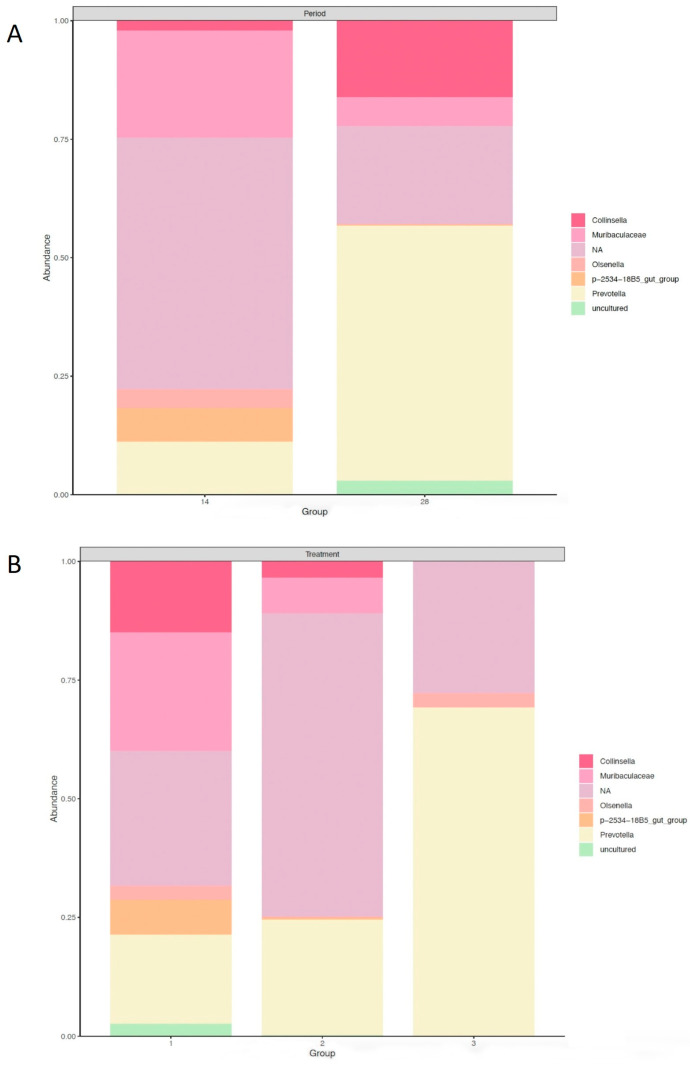
Taxa summary plot by variable. (**A**) Day of postweaning (d 14 and 28 postweaning). (**B**) Dietary treatment (n = 6 pens per treatment). Treatments: (1) control: no probiotic supplementation, (2) Pro1x: *B. subtilis* supplementation at 1.875 × 10^5^ CFU/g diet, and (3) Pro10x: *B. subtilis* supplementation at 1.875 × 10^6^ CFU/g diet.

In the result of the fecal microbiome analysis, a total of 1,249,847 raw reads were obtained from fecal samples collected from 36 animals. After quality control, combining paired-end reads, and filtering chimeras, on average, 72% of sequences passed the filters, and an average of 24,589 (±5186) denoised sequences were generated per animal. The microbial community composition showed differences between d 14 and 28 postweaning and across dietary treatments (Figure 1).

**Figure 2:**
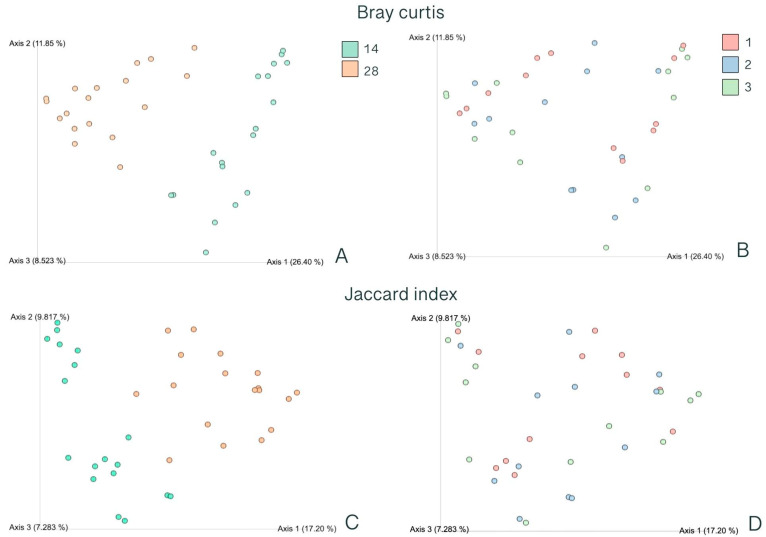
Beta diversity indexes. Bray–Curtis dissimilarity (**A**,**B**) and Jaccard index (**C**,**D**) of fecal samples from swine in two different days of postweaning ((**A**,**C**); n = 18 per day of postweaning) and three different treatments ((**B**,**D**); n = 6 per treatment). (**A**) Bray–Curtis dissimilarity by day of postweaning (d 14 and 28 postweaning). (**B**) Bray–Curtis dissimilarity by dietary treatment. (**C**) Jaccard index by day of postweaning (d 14 and 28 postweaning). (**D**) Jaccard index by dietary treatment. Treatments: (1) control: no probiotic supplementation, (2) Pro1x: *B. subtilis* supplementation at 1.875 × 10^5^ CFU/g diet, and (3) Pro10x: *B. subtilis* supplementation at 1.875 × 10^6^ CFU/g diet.

The day of postweaning and dietary treatment affected the beta diversity of the bacterial community in the feces. The Jaccard index and Bray–Curtis dissimilarity were affected by the day of postweaning (Figure 2A,C). 

**Figure 3:**
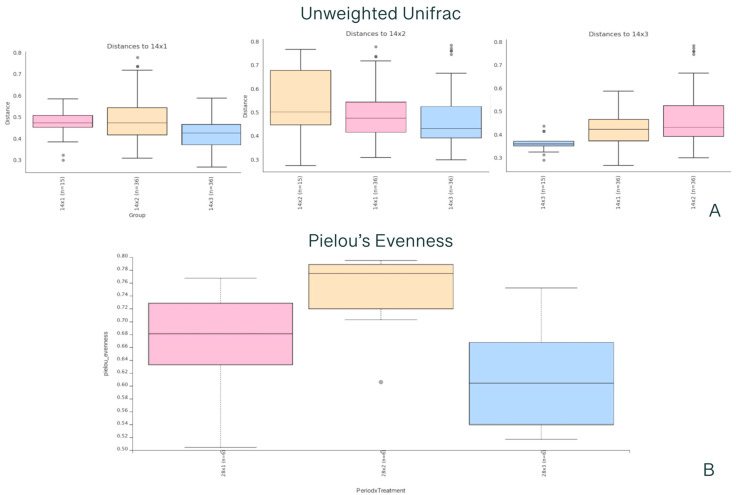
Alpha and beta diversity in feces of nursery pigs with Unweighted Unifrac analysis and Pielou’s Evenness. (**A**) beta diversity based on Unweighted Unifrac analysis at d 14 postweaning and a significant difference (*p* < 0.05) between Pro1x and Pro10x treatments. (**B**) Alpha diversity based on Pielou’s Evenness at d 28 postweaning and a greater evenness (*p* < 0.05) in fecal microbiome in Pro1x treatment than Pro10x treatment. Treatments: (1) control: no probiotic supplementation, (2) Pro1x: *B. subtilis* supplementation at 1.875 × 10^5^ CFU/g diet, and (3) Pro10x: *B. subtilis* supplementation at 1.875 × 10^6^ CFU/g diet. Gray dots are outliers.

There was an interaction of the day of postweaning × dietary treatment, in which a dissimilarity was observed between the Pro1x and Pro10x treatments in beta diversity based on Unweighted Unifrac analysis at d 14 postweaning (*p* < 0.05; Figure 3), and a greater evenness was observed in the Pro1x treatment than in the Pro10x treatment in alpha diversity based on Pielou’s Evenness at d 28 postweaning (*p* < 0.05; Figure 3). Taxonomic profiling revealed a total of four bacterial taxa at the phylum level. The dominant bacterial phylum was Firmicutes (90%), followed by Bacteroidota (9.95%). At the genus level, the predominant taxa were *Lactobacillus* (25%), *Streptococcus* (19%), and *Blautia* (6%) (Supplementary Table S1).

## 4. Discussion

Probiotics have been widely used in swine diets, especially in nursery diets, to improve the gut microbiota and immunity of pigs, thereby reducing postweaning diarrhea and improving postweaning growth performance [2,8]. The efficacy of probiotic products is affected by several factors, such as the species, strains of the microbe, product formulation, health condition, and dose of the product administered, and the effectiveness of dietary probiotic supplementation in nursery diets is inconsistent [8,22]. In addition, there is limited information about the consequences of overdosing probiotic product in nursery pig diets and whether this could still show comparable beneficial effects on pigs. The current study evaluated the effect of an overdose of *B. subtilis* in nursery pig diets compared with treatments with no probiotic supplementation or a recommended dose of probiotic. Although the viable *Bacillus* spp. count was greater than the target level in the Pro10x diet in Phase 2, our analysis confirmed that the piglets consumed diets containing *B. subtilis* above the minimum target level assigned to each treatment.

In the current study, the *B. subtilis* supplementation to nursery pig diets at 1.875 × 10^5^ CFU/g diet in the Pro1x treatment increased the body weight and growth rate in the early postweaning period. This result agrees with previous studies showing that *B. subtilis* supplementation improved nursery pig growth performance. Deng et al. [10] reported that *B. subtilis* supplementation improved growth performance, digestive enzyme activities, and villus/crypt depth ratio in the ileum of nursery pigs. It has also been reported that *B. subtilis* supplementation to nursery pig diets improved the growth performance, gut health, and barrier functions and reduced the severity of diarrhea in weaned pigs challenged with F18 enterotoxigenic *E. coli* [11,12]. In the current study, *B. subtilis* supplementation to the nursery diets at 1.875 × 10^5^ CFU/g diet increased individual SCFA concentrations in feces, including acetate, propionate, butyrate, and isovalerate, at d 28 postweaning, resulting in the greatest total SCFA concentrations in feces among dietary treatments. Previous studies reported that *B. subtilis* supplementation to a weaned pig diet increased SCFA concentrations in the digesta [6,23], which agrees with the current study results. The SCFA and branched-chain fatty acids play important roles in gut integrity as the energy source, nutrient absorption, and gut microbiota in pigs [23,24]. Therefore, the improved growth performance of pigs fed the diets with *B. subtilis* at 1.875 × 10^5^ CFU/g diet may be associated with increased SCFA production in the gut, which could provide additional energy for gut development and growth in piglets. 

The improvement in growth performance early postweaning disappeared in the late nursery period, resulting in no differences in body weight and overall growth rate among dietary treatments. He et al. [12] showed similar results with the current study, in which *B. subtilis* supplementation improved growth performance until d 14 postweaning, but this effect disappeared at d 21 postweaning. Kim et al. [11] reported that increasing levels of *B. subtilis* supplementation improved growth performance until d 11 postinoculation of a pathogenic *E. coli* (19 days postweaning), only with a tendency. This may be related to the health condition of piglets after weaning. The early postweaning period for nursery pigs is critical for newly weaned pigs for their health, growth, and development in the entire nursery period as the low feed intake in the early postweaning period causes morphological and functional changes in the gut, resulting in reduced brush-border enzyme activity and intestinal cell absorptive capacity [25]. As piglets grow after weaning, they recover from gastrointestinal disturbance, diarrhea, and immune stress a few weeks postweaning, although it may take more than 2 weeks for piglets to fully recover their gastrointestinal function and integrity [25]. Therefore, the positive effect of *B. subtilis* supplementation to nursery diets in the early postweaning period indicates that its impact on improving gut health and metabolites in pigs is more pronounced in the early postweaning period than in the late postweaning period.

Interestingly, the *B. subtilis* supplementation in the nursery diets at a level 10 times greater than in the Pro1x treatment neither improved growth performance and fecal SCFA production nor had negative impacts on those parameters, as the growth performance and fecal SCFA concentrations were comparable to those in the control treatment without *B. subtilis* supplementation. In addition, the fecal score was not different among dietary treatments throughout the entire nursery period. Li et al. [26] reported that an overdose of *L. rhamnosus* increased the incidence of diarrhea in weaning pigs before the pathogenic *E. coli* challenge, although it alleviated pathogenic *E. coli*-induced diarrhea and reduced the counts of *Lactobacillus* and *Bacteroides* in the ileal content of weaning pigs. Trevisi et al. [27] reported negative effects of *L. rhamnosus* GG supplementation in pigs challenged with enterotoxigenic *E. coli* F4 on growth performance and fecal score. Therefore, over-dose probiotic supplementation may not be beneficial for weaning pigs, as probiotics theoretically have potential side effects in the immune system, systemic infection, metabolic activities, antibiotic resistance, and the production of harmful metabolites [28]. There are few studies investigating the effect of an overdose of probiotics in humans and pigs, and it has been reported that *B. subtilis* could cause bacteremia in humans and premature infants, and humans with underlying conditions are more susceptible to probiotic sepsis [29]. Based on the current study results, overdosing *B. subtilis* in nursery pig diets (e.g., a 10 times greater supplementation level than in the Pro1x treatment) was neither as effective as its supplementation at 1.875 × 10^5^ CFU/g diet nor detrimental for pigs, although 1.875 × 10^5^ CFU/g diet of *B. subtilis* supplementation showed improved performance and fecal SCFA production. However, this result needs to be interpreted carefully, as no negative impact on the growth performance of nursery pigs does not mean this could be safe for young animals and human infants.

*Bacillus subtilis* supplementation to the nursery diets did not affect blood parameters, including hematocrit and creatinine levels, at d 14 and 28 postweaning, whereas blood glucose concentrations at d 14 postweaning increased through *B. subtilis* supplementation regardless of the supplementation levels. Deng et al. [10] reported that *B. subtilis* supplementation to the diets for nursery pigs at approximately 45 d of age increased serum glucose concentrations at d 28 of the experiment. Similarly, Wang et al. [30] also reported increased plasma glucose levels in pigs at d 21 of the experiment when pigs were fed a *B. subtilis*-supplemented diet from d 25 of age. Mangian and Tappenden [31] reported that butyrate could increase the intestinal absorption of glucose by upregulating glucose transporter 2 mRNA abundance. In the current study, *B. subtilis* supplementation increased butyrate concentrations in the feces, which potentially impacted the glucose absorption of nursery pigs. With low feed intake in the early postweaning period, glucose could be an important energy source in the first few weeks after weaning. Therefore, *B. subtilis* supplementation could be beneficial for weaning pigs as it may increase glucose absorption from the diet.

Despite the nonsignificant effects on fecal bile acid concentrations, subtle influences of *B. subtilis* supplementation on the composition of bile acid pool, as reflected by the increased hyodeoxycholic acid/hyocholic acid ratio and the decrease in glycohyocholic acid on d 14 postweaning under the Pro1x treatment, were observed. Hyocholic acid, a major primary bile acid in pigs, is the direct precursor of hyodeoxycholic acid, an abundant secondary bile acid with diverse bioactivities, including preventing atherosclerosis formation and reducing plasma cholesterol levels in mice [32], and improving glucose homeostasis in pigs [33,34]. The increase in hyodeoxycholic acid/hyocholic acid ratio indicates the increase in microbial metabolism of bile acid, mainly through the increase in bacterial taxa performing 7-α-dehydroxylation reaction [35]. Interestingly, a similar phenomenon of elevated hyodeoxycholic acid has been observed in growing pigs under tylosin treatment, a growth-promoting antibiotic [36], and has been suggested as an indicator of gut microbiome maturation. Further studies are needed to examine how *B. subtilis* probiotics could affect microbial metabolism of bile acids as well as the influences on animal performance.

In the current study, fecal microbial community composition in pigs was different between the early and late postweaning periods, which agrees with previous reports indicating that microbial community changed with age in pigs [37]. Regarding the treatment effects, when their diets were supplemented with *B. subtilis* at a level 10 times greater than in the Pro1x treatment, piglets had dissimilar microbial communities in their feces at d 14 and 28 postweaning. Evenness diversity was lower at d 28 postweaning compared to d 14 postweaning. This result agrees with previous studies, where an alteration in the fecal microbiome in piglets fed various types of *B. subtilis*-containing diets was found [22]. Similarly, Ding et al. [23] reported that *B. subtilis* altered the beta diversity of microbiota in ileum and colon compared with the control treatment. However, several studies failed to alter alpha diversity by feeding *B. subtilis* in feces [38] and in jejunum, ileum, and colon [23]. This result indicates that *B. subtilis* supplementation in nursery pig diets could alter gut microbial community composition, while further studies are needed to dissect the effects of strains, species, supplementation level.

## 5. Conclusions

Dietary *B. subtilis* supplementation of two strains selected to reduce the effects of pathogenic *E. coli* in nursery diets at 1.875 × 10^5^ CFU/g diet improved the growth rate in the early postweaning period, increased major fecal SCFA concentrations such as acetate, propionate, and butyrate that can help the intestinal health of pigs, and altered the ratio between hyodeoxycholic acid and hyocholic acid and the microbial community composition in feces. A higher dose of the probiotic did not improve the measured performance parameters nor the fecal SCFA concentrations over those of the control piglets, although both *B. subtilis* treatments increased the blood glucose levels in the early nursery period. Therefore, when probiotics such as *B. subtilis* are used in diets for weaning pigs, the strains, doses, and other factors need to be considered to maximize the efficacy of their supplementation on the postweaning growth and gut health of the pigs and reduce the feed cost, as an overdose of probiotics could be redundant. In addition, a larger-scale (commercial farm-size) feeding study may be needed to clearly demonstrate its effectiveness in actual pig farming.

## Figures and Tables

**Table 1:** Diet formulation and calculated chemical composition ^1^.

Ingredients, %	Phase 1 d 0–14 Postweaning	Phase 2 d 15–28 Postweaning
Corn	49.25	56.75
Soybean meal (48% CP)	20.00	25.00
Fish meal	2.25	1.25
Blood meal	2.50	0.00
Whey, dried	15.00	6.25
Oats	2.50	2.50
Soy oil	1.85	1.50
Molasses	1.50	1.75
L-Lysine·HCl	0.15	0.00
Trace mineral and vitamin premix ^2^	5.00	5.00
Calculated chemical composition		
Metabolizable energy (kcal/kg)	3350	3310
Crude protein (%)	20.32	19.27
SID ^3^ lysine (%)	1.42	1.24
SID methionine + cysteine (%)	0.78	0.76
Total Ca (%)	1.01	0.90
Total P (%)	0.68	0.64

^1^ Probiotic product was replaced with corn to mix each treatment diet. ^2^ The trace mineral and vitamin premix supplied the following per kilogram of diet: 53 mg of Mn as manganese sulfate, 150 mg of Fe as ferrous sulfate, 300 mg of Zn as zinc sulfate, 240 mg of Cu as copper sulfate, 0.9 mg of I as ethylenediamine dihydroiodide, 0.36 mg of Se as sodium selenite with 0.48% salt, 13,200 IU of vitamin A, 2112 IU of vitamin D_3_, 158 IU of vitamin E, 2.6 mg of vitamin K, 42.2 mg of vitamin B_12_, 12.0 mg of riboflavin, 79 mg of pantothenic acid, 60 mg of niacin, 1.6 mg of folic acid, 3.4 mg of vitamin B_6_, 2.4 mg of thiamin, and 0.11 mg of biotin. ^3^ SID = standardized ileal digestible.

**Table 2:** Enumeration of *Bacillus* spp. in experimental diets.

	Treatment ^1^		
	Control	Pro1x	Pro10x
Phase 1, CFU/g diet			
Target	-	1.875 × 10^5^	1.875 × 10^6^
Actual	1.20 × 10^4^	1.80 × 10^5^	2.20 × 10^6^
Phase 2, CFU/g diet			
Target	-	1.875 × 10^5^	1.875 × 10^6^
Actual	2.80 × 10^4^	1.70 × 10^5^	6.70 × 10^6^

^1^ Treatments were as follows: (1) control: no probiotics supplementation, (2) Pro1x: *B. subtilis* supplementation at 1.875 × 10^5^ CFU/g diet, and (3) Pro10x: *B. subtilis* supplementation at 1.875 × 10^6^ CFU/g diet.

**Table 3:** Blood parameters of pigs fed the diets with different levels of *Bacillus subtilis* after weaning ^1^.

	Treatment ^2^				
	Control	Pro1x	Pro10x	SEM ^3^	*p*-Value
Hematocrit, %					
d 14 postweaning	31.2	31.2	31.7	1.13	0.94
d 28 postweaning	35.4	37.2	36.4	1.20	0.48
Glucose, mg/dL					
d 14 postweaning	95.2 ^b^	111.7 ^a^	114.3 ^a^	5.15	0.05
d 28 postweaning	114.2	112.7	115.3	4.07	0.90
Creatinine, mg/dL					
d 14 postweaning	0.87	0.80	0.85	0.06	0.56
d 28 postweaning	0.90	0.93	0.82	0.04	0.12

^a,b^ Means within the same row with different superscripts differ (*p* < 0.05). ^1^ n = 6 individual pigs per treatment. ^2^ Treatments were as follows: (1) control: no probiotic supplementation, (2) Pro1x: *B. subtilis* supplementation at 1.875 × 10^5^ CFU/g diet, and (3) Pro10x: *subtilis* supplementation at 1.875 × 10^6^ CFU/g diet. ^3^ SEM, standard error of the mean.

**Table 4:** Fecal short-chain fatty acid concentrations of pigs fed the diets with different levels of *Bacillus subtilis* after weaning ^1^.

	Treatment ^2^				
	Control	Pro1x	Pro10x	SEM ^3^	*p*-Value
D 14 postweaning, µmol/g feces					
Acetate	121.08	139.18	130.10	9.59	0.44
Propionate	63.80	69.10	76.48	8.12	0.56
Butyrate	30.87	42.10	44.08	6.40	0.28
Isovalerate	2.58	3.70	2.68	0.65	0.38
Valerate	13.40	16.47	18.10	3.35	0.62
Total	231.77	270.52	271.50	26.72	0.51
D 28 postweaning, µmol/g feces					
Acetate	120.02 ^ab^	141.87 ^a^	101.17 ^b^	10.71	0.02
Propionate	62.95 ^ab^	71.42 ^a^	55.27 ^b^	7.70	0.03
Butyrate	36.17^b^	47.57 ^a^	33.23 ^b^	5.30	0.04
Isovalerate	2.67 ^b^	3.93 ^a^	2.10 ^b^	0.40	0.01
Valerate	14.95	17.42	14.02	2.87	0.40
Total	236.72 ^ab^	282.23 ^a^	205.80 ^b^	25.62	0.02

^a,b^ Means within the same row with different superscripts differ (*p* < 0.05). ^1^ n = 6 individual pigs per treatment. ^2^ Treatments were as follows: (1) control: no probiotic supplementation, (2) Pro1x: *B. subtilis* supplementation at 1.875 × 10^5^ CFU/g diet, and (3) Pro10x: *B. subtilis* supplementation at 1.875 × 10^6^ CFU/g diet. ^3^ SEM, standard error of the mean.

**Table 5:** Fecal bile acid concentrations of pigs fed the nursery diets with different levels of *Bacillus subtilis* after weaning (d 14 postweaning) ^1^.

	Treatment ^2^				*p*-Value
	Control	Pro1x	Pro10x	SEM ^3^	
Bile acid concentrations, µg/g feces					
Cholic acid	0.39	0.40	0.35	0.11	0.93
Chenodeoxycholic acid	6.91	9.05	8.94	3.73	0.87
Deoxycholic acid	1.15	2.68	1.45	0.94	0.38
Lithocholic acid	143.80	247.50	227.43	43.68	0.25
Hyodeoxycholic acid	493.74	598.93	759.31	141.03	0.44
Hyocholic acid	264.65	120.11	129.35	97.78	0.53
Glycohyodeoxycholic acid	5.48	6.54	5.89	2.49	0.96
Glycohyocholic acid	3.63	1.90	0.93	0.93	0.17
Glycochenodeoxycholic acid	0.93	1.01	0.91	0.34	0.97
Total	920.68	988.11	1134.53	218.48	0.76
Bile acid composition, % of total bile acid					
Cholic acid	0.05	0.05	0.03	0.01	0.64
Chenodeoxycholic acid	0.72	0.67	0.91	0.26	0.77
Deoxycholic acid	0.13	0.20	0.12	0.04	0.20
Lithocholic acid	17.95	31.11	20.20	5.76	0.27
Hyodeoxycholic acid	51.03	58.99	68.54	7.54	0.30
Hyocholic acid	28.62	7.83	9.40	7.47	0.14
Glycohyodeoxycholic acid	0.81	0.86	0.62	0.29	0.83
Glycohyocholic acid	0.55 ^a^	0.15 ^b^	0.10 ^b^	0.15	0.09
Glycochenodeoxycholic acid	0.13	0.14	0.08	0.04	0.46
Ratio of secondary bile acid/primary bile acid					
Hyodeoxycholic acid/hyocholic acid	14.64 ^b^	59.58 ^a^	31.67 ^ab^	15.32	0.08
Lithocholic acid/chenodeoxycholic acid	35.10	80.32	48.14	22.76	0.37

^a,b^ Means within the same row with different superscripts differ (*p* < 0.10). ^1^ n = 6 individual pigs per treatment. ^2^ Treatments were as follows: (1) control: no probiotic supplementation, (2) Pro1x: *B. subtilis* supplementation at 1.875 × 10^5^ CFU/g diet, and (3) Pro10x: *B. subtilis* supplementation at 1.875 × 10^6^ CFU/g diet. ^3^ SEM, standard error of the mean.

**Table 6:** Fecal bile acid concentrations of pigs fed the nursery diets with different levels of *Bacillus subtilis* after weaning (d 28 postweaning) ^1^.

	Treatment ^2^				*p*-Value
	Control	Pro1x	Pro10x	SEM ^3^	
Bile acid concentrations, µg/g feces					
Cholic acid	0.43	0.57	0.49	0.10	0.65
Chenodeoxycholic acid	18.82	6.23	8.94	5.68	0.29
Deoxycholic acid	1.08	0.84	0.68	0.16	0.26
Lithocholic acid	214.70	213.40	183.17	25.30	0.57
Hyodeoxycholic acid	890.00	717.23	954.85	109.64	0.30
Hyocholic acid	35.73	6.30	25.88	12.42	0.17
Glycohyodeoxycholic acid	7.03	7.70	5.62	2.04	0.73
Glycohyocholic acid	1.38	1.16	0.82	0.55	0.77
Glycochenodeoxycholic acid	1.06	1.05	0.75	0.18	0.41
Total	1170.20	954.51	1181.17	126.06	0.37
Bile acid composition, % of total bile acid					
Cholic acid	0.05	0.06	0.04	0.01	0.55
Chenodeoxycholic acid	1.35	0.67	0.81	0.33	0.34
Deoxycholic acid	0.09	0.09	0.06	0.01	0.25
Lithocholic acid	19.45	22.68	16.41	2.46	0.22
Hyodeoxycholic acid	75.52	74.74	79.89	2.04	0.13
Hyocholic acid	2.65	0.65	2.13	0.92	0.18
Glycohyodeoxycholic acid	0.67	0.86	0.52	0.25	0.39
Glycohyocholic acid	0.12	0.13	0.08	0.06	0.72
Glycochenodeoxycholic acid	0.10	0.11	0.07	0.02	0.27
Ratio of secondary bile acid/primary bile acid					
Hyodeoxycholic acid/hyocholic acid	89.34	297.23	79.51	101.49	0.28
Lithocholic acid/chenodeoxycholic acid	25.55	73.42	22.79	22.24	0.24

^1^ n = 6 individual pigs per treatment. ^2^ Treatments were as follows: (1) control: no probiotic supplementation, (2) Pro1x: *B. subtilis* supplementation at 1.875 × 10^5^ CFU/g diet, and (3) Pro10x: *B. subtilis* supplementation at 1.875 × 10^6^ CFU/g diet. ^3^ SEM, standard error of the mean.

## Data Availability

The data in the present study are available from the corresponding author upon reasonable request.

## Supplementary Materials

The following supporting information can be downloaded at: https://www.mdpi.com/article/10.3390/ani14010109/s1, Table S1: Microbial composition on the feces of pigs with the diet supplemented with two strains of *Bacillus subtilis.*
